# Synthesis of Sulfur Vacancy-Bearing In_2_S_3_/CuInS_2_ Microflower Heterojunctions via a Template-Assisted Strategy and Cation-Exchange Reaction for Photocatalytic CO_2_ Reduction

**DOI:** 10.3390/molecules29143334

**Published:** 2024-07-16

**Authors:** Aizhen Liao, Zhengchu Liu, Yiqing Wei, Qinghua Xie, Ting Kong, Maolin Zeng, Wenpeng Wang, Chao Yang, Linji Zhang, Yonggang Xu, Yong Zhou, Zhigang Zou

**Affiliations:** 1 School of Science, Xi’an University of Posts and Telecommunications, Xi’an 710121, China; xqh1115@stu.xupt.edu.cn (Q.X.); kongting0302@xupt.edu.cn (T.K.); zml@stu.xupt.edu.cn (M.Z.); wangwenpeng@xupt.edu.cn (W.W.); yang_chaomail@163.com (C.Y.); zhanglinji@xupt.edu.cn (L.Z.); ygxu@xupt.edu.cn (Y.X.); 2 National Laboratory of Solid State Microstructures, Collaborative Innovation Center of Advanced Microstructures, School of Physics, Nanjing University, Nanjing 210093, China; mf1922005@smail.nju.edu.cn (Z.L.); dg20220060@smail.nju.edu.cn (Y.W.); zgzou@nju.edu.cn (Z.Z.); 3 Ecomaterials and Renewable Energy Research Center, School of Physics, Nanjing University, Nanjing 210093, China

**Keywords:** Vs-In_2_S_3_/CuInS_2_ heterojunctions, flower-like microspheres, template-assisted strategy, cation exchange, photocatalytic CO_2_ reduction

## Abstract

The synthesis of the accurate composition and morphological/structural design of multielement semiconductor materials is considered an effective strategy for obtaining high-performance hybrid photocatalysts. Herein, sulfur vacancy (Vs)-bearing In_2_S_3_/CuInS_2_ microflower heterojunctions (denoted Vs-In_2_S_3_/CuInS_2_) were formed in situ using In_2_S_3_ microsphere template-directed synthesis and a metal ion exchange-mediated growth strategy. Photocatalysts with flower-like microspheres can be obtained using hydrothermally synthesized In_2_S_3_ microspheres as a template, followed by Ostwald ripening growth during the metal cation exchange of Cu^+^ and In^3+^. The optimal heterostructured Vs-In_2_S_3_/CuInS_2_ microflowers exhibited CO and CH_4_ evolution rates of 80.3 and 11.8 μmol g^−1^ h^−1^, respectively, under visible-light irradiation; these values are approximately 4 and 6.8 times higher than those reported for pristine In_2_S_3_, respectively. The enhanced photocatalytic performance of the Vs-In_2_S_3_/CuInS_2_ catalysts could be attributed to the synergistic effects of the following factors: (i) the constructed heterojunctions accelerate charge-carrier separation; (ii) the flower-like microspheres exhibit highly uniform morphologies and compositions, which enhance electron transport and light harvesting; and (iii) the vs. may trap excited electrons and, thus, inhibit charge-carrier recombination. This study not only confirms the feasibility of the design of heterostructures on demand, but also presents a simple and efficient strategy to engineer metal sulfide photocatalysts with enhanced photocatalytic performance.

## 1. Introduction

The photocatalytic reduction of CO_2_ into carbonaceous fuels has attracted much attention because it offers a feasible strategy for reducing the consumption of fossil fuels and preventing the emission of greenhouse gases [1,2,3,4]. Various types of semiconductor-based photocatalysts, such as TiO_2_, BiVO_4_, In_2_O_3_, and WO_3_, have been employed to transform CO_2_ into high-value fuels, such as CO, CH_3_OH, HCOOH, and CH_4_ [5,6,7]. However, the performance of these photocatalysts is limited by their insufficient active sites, low band gap energies, and high charge-carrier recombination rate. Hence, the elaborate design of advanced photocatalysts with outstanding performance and excellent stability is of great importance.

Metal sulfides are fascinating photocatalytic materials because they possess excellent photosensitivity, low redox potential, appropriate band edges, moderate band potential, high stability, good charge-carrier mobility, etc. In_2_S_3_ and CuInS_2_, in particular, have emerged as promising catalysts owing to their visible-light response and appropriate band gaps (In_2_S_3_: 2–2.3 eV; CuInS_2_: 1.57 eV) for CO_2_ conversion [8,9,10,11]. For example, In_2_S_3_-based photocatalysts, such as In_2_S_3_/In_2_O_3_/rGO [7], Bi_2_S_3_@In_2_S_3_ [12], and In_2_S_3_/C_3_N_4_ [13], can transform CO_2_ into CO, CH_4_, or C_2_H_4_. CuInS_2_-based photocatalysts, such as Ti_3_C_2_ MXene@TiO_2_/CuInS_2_ [14], ZnIn_2_S_4_/CuInS_2_ [15], and CuInS_2_/ZnS [16], also exhibit photocatalytic activity for the reduction of CO_2_ to CO and H_2_. Heterojunctions have recently been demonstrated to maximize the performance of In_2_S_3_/CuInS_2_ photocatalysts and increase their ability for CO_2_ reduction [17,18]. However, the synthesis of the accurate composition and morphological/structural design of multielement CuInS_2_ semiconductor materials on the surface of In_2_S_3_ is challenging because of the difficulty of balancing the various reactivities of multiple reactants. Several studies have pointed out that the accurate composition and morphological/structural design of semiconductors are effective strategies for obtaining photocatalysts with outstanding performance and excellent stability [19,20]. Thus, further explorations of suitable strategies for designing special morphological In_2_S_3_/CuInS_2_ photocatalysts are necessary.

Photocatalysts with three-dimensional flower-like structures are intriguing materials that have recently shown great potential for photocatalytic CO_2_ reduction because of their intrinsic properties, which include a large surface area enriched with exposed active sites, which promotes CO_2_ capture and conversion catalysis, a lessened perpendicular charge-transfer distance, which prevents electron (e^−^)–hole (h^+^) recombination, and internal reflection/dispersion effects, which reinforce light utilization [21,22]. However, the synthesis of flower-like micro/nanocrystals is challenging because it requires the refined tuning of nucleation, growth of particles, and surface binding dynamics for selective crystal face exposure. Hence, common strategies to prepare semiconducting flower-like micro/nanocrystals with controllable compositions and structures are highly desirable for achieving outstanding photocatalysts for carbonaceous fuel production.

Cation-exchange reactions, in which the bonding cations of the host sublattice are replaced by external mobile cations, present a promising method for fabricating hollow photocatalysts based on the Kirkendall effect [23,24]. For instance, using the Kirkendall effect driven by cation exchange between Cu^+^ and In^3+^, Li et al. synthesized controllable compositions and morphology/structures of hollow CuInS_2_ nanododecahedrons [25]. This type of cation exchange not only allows for multiple cations to be wholly or partly replaced with each other, but also achieves powerful synthetic control of the composition of the desired materials. Furthermore, semiconductor structures preserve the anisotropic shapes of the as-prepared templates. Wang et al. prepared various hollow multinary metal sulfides, including binary compounds (CdS, ZnS, Ag_2_S, PbS, SnS), ternary compounds (CuInS_2_, Zn*_x_*Cd_1−*x*_S), and quaternary compounds (single-atom Pt-anchored Zn*_x_*Cd_1−*x*_S, Zn*_x_*Cd_1−*x*_S-Pt_1_), using Cu_2−*x*_S nanocubes as the initial template. In this case, Cu^+^ was replaced by cations, the Cu_2−*x*_S nanocube framework was conserved, and precise control of distinct elementary compositions was achieved by adjusting the ratio and type of the metal cation source, all of which significantly improved the photocatalytic performance of the resulting catalysts [26]. It has been argued that this is a simple but efficient approach to attain precise compositions and morphological/structural of multicomponent semiconductor materials.

In this work, sulfur vacancy (Vs)-bearing In_2_S_3_/CuInS_2_ with microflower heterojunctions (denoted Vs-In_2_S_3_/CuInS_2_) were formed in situ using an In_2_S_3_ microsphere template-directed synthesis and metal ion exchange reaction. Hybrid photocatalysts with flower-like microspheres were obtained using hydrothermally synthesized In_2_S_3_ microspheres as a template, followed by Ostwald ripening growth during the metal cation exchange of Cu^+^ and In^3+^. The optimal heterostructured Vs-In_2_S_3_/CuInS_2_ microflowers exhibited excellent activity, with CO and CH_4_ evolution rates of 80.3 and 11.8 μmol g^−1^ h^−1^, respectively, under visible-light irradiation; these values are roughly 4 and 6.8 times higher than those reported for pristine In_2_S_3_, respectively. The enhanced photocatalytic performance of the Vs-In_2_S_3_/CuInS_2_ catalysts can be attributed to the synergistic effects of the following factors: (i) the constructed heterojunctions accelerate charge-carrier separation; (ii) the flower-like microspheres exhibit highly uniform morphologies and compositions, which enhance electron transport and light harvesting; and (iii) the vs. may trap excited electrons and, thus, inhibit charge-carrier recombination.

## 2. Results and Discussion

**Figure 1:**
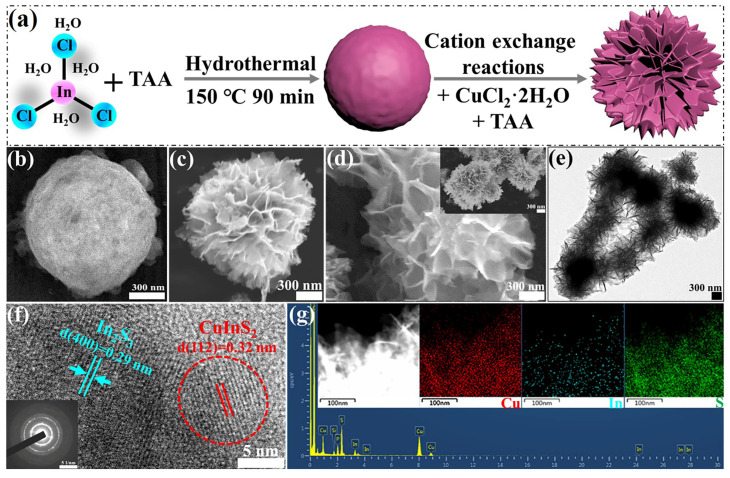
(**a**) Schematic illustration of the synthetic process of the Vs-In_2_S_3_/CuInS_2_ composites; (**b**) SEM image of In_2_S_3_; (**c**,**d**) SEM, (**e**) TEM, (**f**) HRTEM, and (**g**) TEM-EDS images of Vs-In_2_S_3_/CuInS_2_. The insets in (**d**,**f**) show the diminished image of (**d**) and corresponding SAED pattern of the composite.

Figure 1a shows a schematic of the synthetic process for the Vs-In_2_S_3_/CuInS_2_ composites by using an In_2_S_3_ microsphere template-assisted and cation exchange-mediated growth strategy. Typically, In_2_S_3_ template materials with controllable morphology are first synthesized by a facile low-temperature solvothermal method. The mixed solution, including an appropriate amount of the preprepared In_2_S_3_, CuCl_2_·2H_2_O, and thioacetamide (TAA), is then heated. The SEM image in Figure 1b shows that the as-prepared In_2_S_3_ has a microspherical structure with a rough and uniform surface. The spherical architectures are composed of small nanoparticles, and the average diameter of all the spheres is in the range of 1–2 μm. Figure 1c,d show SEM images of Vs-In_2_S_3_/CuInS_2_, while Figure 1e shows its TEM image. The images reveal that the as-derived Vs-In_2_S_3_/CuInS_2_ maintains the spherical shape of the In_2_S_3_ microsphere template and that all of the spheres have a uniform morphology with an average diameter of 1–2 μm. However, the surfaces of the microspheres exhibit numerous flakes (thickness, <15 nm) aligned with each other. The unique morphology of Vs-In_2_S_3_/CuInS_2_ may be derived from Ostwald ripening growth during the metal cation exchange of Cu^+^ and In^3+^. The HRTEM image shown in Figure 1f reveals lattice spacings of 0.32 and 0.29 nm, which correspond to the (112) crystal plane of CuInS_2_ and the (400) crystal plane of In_2_S_3_, respectively; these findings imply that CuInS_2_ is orthotopically grown on the surface of the In_2_S_3_ microspheres, leading to the successful preparation of Vs-In_2_S_3_/CuInS_2_. The selected area electron diffraction (SAED; Figure 1f, inset) pattern of the Vs-In_2_S_3_/CuInS_2_ composite shows that the flower-like microspheres are polycrystalline, indicating that CuInS_2_ is generated along with Ostwald ripening growth spatial orientation during the cation-exchange process; this finding is consistent with the TEM results. The elemental composition of the composites, which includes Cu, In, and S, is confirmed by the elemental mappings shown in Figure 1g.

**Figure 2:**
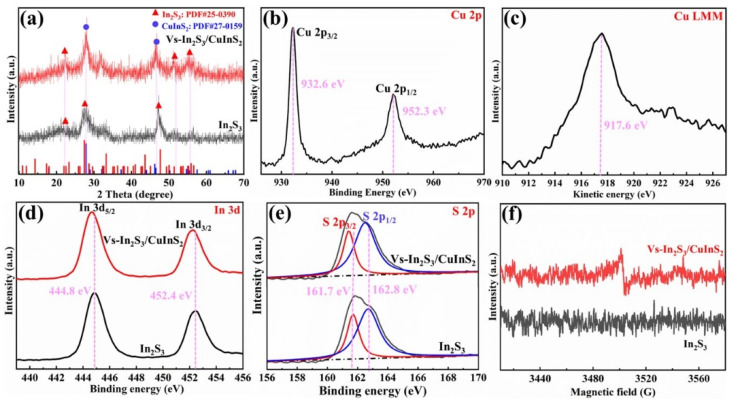
(**a**) XRD patterns of In_2_S_3_ and Vs-In_2_S_3_/CuInS_2_; (**b**–**e**) high-magnification XPS profiles of (**b**) Cu 2p, (**c**) Cu LMM, (**d**) In 3d, and (**e**) S 2p; (**f**) EPR signals of In_2_S_3_ and Vs-In_2_S_3_/CuInS_2_.

The XRD patterns shown in Figure 2a reveal that all the diffraction peaks of In_2_S_3_ can be well indexed to the standard card (JCPDS No. 25-0390), and no other phase is observed (Figure 2a). Furthermore, most of the diffraction peaks in the XRD pattern of Vs-In_2_S_3_/CuInS_2_ can be indexed to the composite phase of In_2_S_3_ and CuInS_2_ (In_2_S_3_: JCPDS No. 25-0390; CuInS_2_: JCPDS No. 27-0159).

XPS was used to investigate the elemental composition and valence environment of the samples. The survey XPS profile of Vs-In_2_S_3_/CuInS_2_ reveals the existence of Cu, In, and S (Figure S1). The two peaks in the Cu 2p spectrum (Figure 2b) with binding energies of 932.6 and 952.3 eV are assigned to Cu 2p_3/2_ and Cu 2p_1/2_ of Cu^+^, respectively. These peaks coincide with the literature description of the valence state of Cu in CuInS_2_, and verify the successful growth of CuInS_2_ on In_2_S_3_. The peak in the Cu LMM (Auger electron) spectrum (Figure 2c) at 917.6 eV reveals that the oxidation number of Cu is +1. The peaks in the XPS profile of In 3d (Figure 2d) at 444.8 and 452.4 eV are ascribed to the core lines of the In–S bond. The binding energy of In 3d in In_2_S_3_ is 0.10 eV lower than that in Vs-In_2_S_3_/CuInS_2_ owing to the existence of CuInS_2_. The S 2p spectra of In_2_S_3_ and Vs-In_2_S_3_/CuInS_2_ can be deconvoluted into two peaks with binding energies of 162.8 and 161.7 eV, which are consistent with S^2−^ in the crystal lattice. The binding energy of S 2p in In_2_S_3_ is 0.10 eV lower than that in Vs-In_2_S_3_/CuInS_2_, probably because of the existence of vs. or CuInS_2_ (Figure 2e). The presence of vs. was further confirmed by EPR spectroscopy. Several vs. are formed on the In_2_S_3_/CuInS_2_ surface because it is S-deficient owing to the sharing of S atoms (Figure 2f). Numerous studies have shown that vs. defects can not only regulate the electronic structure/state of a photocatalyst but also serve as active sites to adsorb and activate CO_2_ molecules, thus leading to improved photoactivity [27,28].

**Figure 3:**
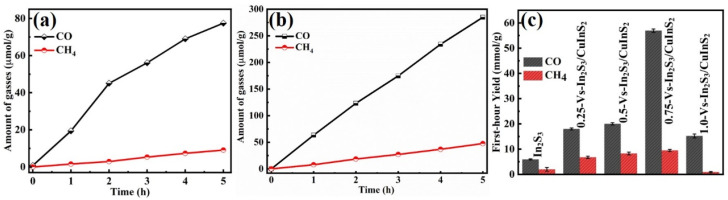
(**a**,**b**) Amounts of CO and CH_4_ formed over (**a**) In_2_S_3_ and (**b**) 0.75-Vs-In_2_S_3_/CuInS_2_ as a function of the light irradiation time; (**c**) comparison of the photocatalytic activities of In_2_S_3_, 0.25-Vs-In_2_S_3_/CuInS_2_, 0.50-Vs-In_2_S_3_/CuInS_2_, 0.75-Vs-In_2_S_3_/CuInS_2_, and 1.0-Vs-In_2_S_3_/CuInS_2_ for the first hour.

Photocatalytic CO_2_ conversion was performed in the presence of water vapor under simulated solar irradiation (Figure 3). CO as the major product and a small amount of CH_4_ as the minor product were detected over In_2_S_3_ and Vs-In_2_S_3_/CuInS_2_. In_2_S_3_ could produce 20 μmol g^−1^ CO and 1.73 μmol g^−1^ CH_4_ after 1 h (Figure 3a), and these yields gradually increase with increasing irradiation time. Compared with that of In_2_S_3_, the photocatalytic activity of Vs-In_2_S_3_/CuInS_2_ is significantly improved, and the yields of CO and CH_4_ over the catalyst gradually increase as the amount of CuCl_2_·2H_2_O increases from 0.25 to 0.75 g (Figure 3b). Maximum yields of 80.3 μmol g^−1^ CO and 11.8 μmol g^−1^ CH_4_ are achieved over 0.75-Vs-In_2_S_3_/CuInS_2_ within 1 h; these yields are 4 and 6.8 times greater than those produced over In_2_S_3_, respectively. However, the use of 1.0 g of CuCl_2_·2H_2_O leads to a reduction in activity, possibly because excess vs. defects may accelerate the recombination of photogenerated carriers (Figure 3c). The enhanced photocatalytic performance of the Vs-In_2_S_3_/CuInS_2_ composite could be attributed to its flower-like microsphere structure, which features a large surface area that could promote the exposure of active sites and increase charge separation owing to the short charge-transport distance from the bulk to the surface. Moreover, the existence of vs. may trap excited electrons, thereby inhibiting charge-carrier recombination.

**Figure 4:**
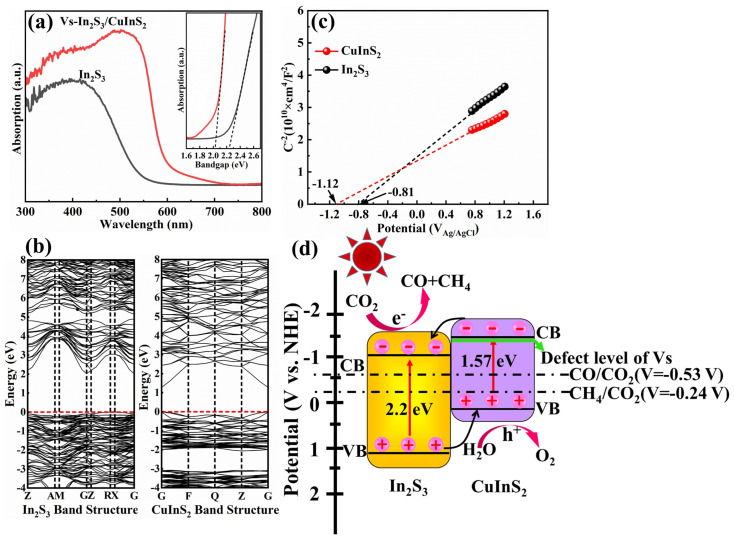
(**a**) UV−visible absorption spectrum of Vs−In_2_S_3_/CuInS_2_ (inset: estimated band gap energy of Vs−In_2_S_3_/CuInS_2_ based on its absorption); (**b**) calculated band gap energies for In_2_S_3_ and CuInS_2_; (**c**) Mott–Schottky plots for In_2_S_3_ and CuInS_2_; and (**d**) energy band structure of In_2_S_3_, CuInS_2_ and Vs illustration of charge transfer at the Vs−In_2_S_3_/CuInS_2_ heterojunction.

Considering that the photocatalytic behavior of semiconductors is closely related to their optical properties, UV-visible absorption spectroscopy was conducted on the samples (Figure 4a). Vs-In_2_S_3_/CuInS_2_ shows significant absorption over nearly the entire visible region up to 700 nm. Its corresponding band gap energy is estimated to be 2.02 eV (Figure 4a, inset), which is lower than that of pure In_2_S_3_ (2.2 eV) owing to the low band gap energy of CuInS_2_. The calculated band gap energy of CuInS_2_ is 1.2 eV (Figure 4b, right), which approximately matches the literature value of 1.57 eV [11]. Theoretical calculations indicate that the band gap energy of In_2_S_3_ is approximately 2.13 eV, which closely matches the experimental result (Figure 4b, left). These results illustrate the reliability of the experimental data. Additionally, the absorption intensity of Vs-In_2_S_3_/CuInS_2_ is improved compared with that of In_2_S_3_ because of its flower-like microsphere structure. The flat band potentials (E_FB_) of the samples were acquired from the tangent of their Mott–Schottky curves at a frequency of 1000 Hz (Figure 4c). The E_FB_ values of In_2_S_3_ and CuInS_2_ are −0.81 and −1.12 eV, respectively. In general, the E_FB_ is approximately 0.2 eV more positive than the conduction band edge (E_CB_) of n-type semiconductors. Thus, the E_CB_ values of In_2_S_3_ and CuInS_2_ can be estimated to be −1.01 and −1.32 eV, respectively. According to the equation E_VB_ = E_CB_ + E_g_, the minimum positions of the corresponding conduction bands (CBs) of In_2_S_3_ and CuInS_2_ may occur at 1.19 and 0.25 eV, respectively. The energy band structures of In_2_S_3_ and CuInS_2_ are illustrated in Figure 4d; the well-aligned bands shown in the figure meet the photocatalytic CO_2_ reduction potential. Under light irradiation, the electrons are excited from the VB to the CBs of In_2_S_3_ and CuInS_2_. Owing to the formation of heterojunctions, the excited electrons of CuInS_2_ transfer to the CBs of In_2_S_3_. The photoinduced electrons then react with CO_2_ to form CO and CH_4_. Simultaneously, the photoinduced holes could transfer from the valence band of the In_2_S_3_ to that of CuInS_2_. NaSO_3_, as the sacrificial agent, reacts with holes to further inhibit the recombination of photoinduced electrons and holes [29]. Moreover, previous work has reported that when vacancies are generated, a defect energy level will form in the forbidden band and the higher the sulfur vacancy concentration, the closer the defect level is to the CB position [27]. The EPR test (Figure 2d) verified that Vs exist in the In_2_S_3_/CuInS_2_ sample; therefore, a defect energy level forms below the CBs of CuInS_2_ (Figure 4d), which could easily trap the photogenerated charge carriers.

**Figure 5:**
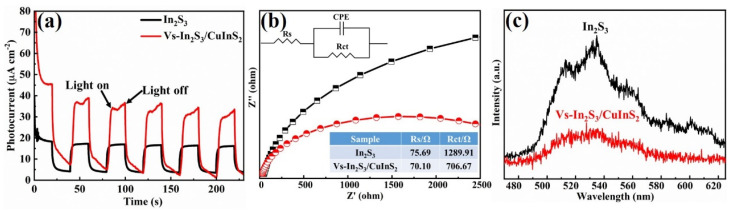
(**a**) Photocurrent measurements of In_2_S_3_ and 0.75-Vs-In_2_S_3_/CuInS_2_; (**b**) Nyquist plots of In_2_S_3_ and Vs-In_2_S_3_/CuInS_2_ (here, the symbols and solid lines represent the experimental data and fitting results, respectively (inset: equivalent circuit model for data fitting)); and (**c**) the steady-state PL spectra of In_2_S_3_ and Vs-In_2_S_3_/CuInS_2_.

Figure 5a shows the transient photocurrent measurements of the as-prepared In_2_S_3_ and Vs-In_2_S_3_/CuInS_2_ photoelectrodes under intermittent light irradiation; here, the light was switched on and off at 250 s intervals. The transient photocurrent for Vs-In_2_S_3_/CuInS_2_ is higher than that for In_2_S_3_, indicating a higher charge-separation efficiency of the photogenerated carriers when using the Vs-In_2_S_3_/CuInS_2_ composite. This result implies that the formation of a flower-like microsphere structure and heterojunctions enhance the separation efficiency of photoinduced charges, leading to improved photocatalytic performance. The Nyquist plots shown in Figure 5b reveal that the extent of photocurrent enhancement exhibited by Vs-In_2_S_3_/CuInS_2_ could be correlated with the reduction in the charge-transfer resistance of the material. Fitting the experimental data to an equivalent resistance/capacitance circuit model (Figure 4a, inset) reveals that compared with In_2_S_3_, Vs-In_2_S_3_/CuInS_2_ presents lower charge-transfer resistance, indicating easier charge transfer on the catalyst surface owing to the existence of a flower-like microsphere structure, Vs, and heterojunctions in the composite. The room-temperature steady-state PL of the developed catalyst was further investigated to understand its photogenerated charge-transfer mechanism. The intensity of the room-temperature steady-state PL peak of Vs-In_2_S_3_/CuInS_2_ is weaker than that of In_2_S_3_ (Figure 5c), indicating that the recombination efficiency of the photogenerated electrons and holes is low. Based on the above analyses, the enhanced photocatalytic performance of the Vs-In_2_S_3_/CuInS_2_ catalysts could be attributed to the synergistic effect of the following factors: (i) the formation of a flower-like microsphere structure with high morphological and compositional uniformity, which enhances light harvesting and electron transport; (ii) the acceleration of charge-carrier separation by the constructed heterojunctions; and (iii) the introduction of Vs, which may trap excited electrons and inhibit charge-carrier recombination.

To reveal the underlying reasons for the improved CO_2_ photoreduction performances over the Vs-In_2_S_3_/CuInS_2_ heterostructure, a possible mechanism of selective photocatalytic CO_2_ reduction is proposed. Prior reports [30,31] indicate that the selectivity of methane during CO_2_ reduction is strongly related to the reaction path of CO* to CHO*, which determines whether the CO* intermediates desorb to CO (g) or continue protonation to CHO* to yield CH_4_. Xie et al. [31] report that the reaction energy from CO* to CHO* of the In_2_S_3_ slab is much higher than that from CO* to CO (g), consistent with the low activity towards CH_4_ during CO_2_ photoreduction in Figure 3a. Compared with that of In_2_S_3_, the photocatalytic activity of Vs-In_2_S_3_/CuInS_2_ is significantly improved, its products are still CO and CH_4_, and the yields of CO always exceed those of CH_4_ (Figure 3b). These results suggest that the reaction energy from CO* to CO (g) and CO* to CHO* of the Vs-In_2_S_3_/CuInS_2_ heterostructure slab was distinctly lower than In_2_S_3_, which could be attributed to the optimized carrier dynamics on the hybrid photocatalyst slab, including the generation and separation of the carrier. Furthermore, vs. may act as adsorption sites for CO_2_ [28], conducive to the further hydrogenation of the CO intermediate into CH_4_. To understand the deep behavior of the photocatalytic reduction of CO_2_, this aspect needs to be further studied in the future.

In short, this study offers a significant contribution to the literature because we not only confirmed the feasibility of the design of heterostructures on demand, but also demonstrated the benefits of such heterostructures for improving the photochemical properties of In_2_S_3_-based photocatalysts. However, satisfactory photocatalytic efficiency in applications such as solar energy conversion, environmental remediation, chemosensors, etc., has not been achieved until now. Thus, intensive investigations should be carried out in the future, in order to provide scientific references for maximizing the photocatalytic efficiencies of sulfide semiconductors.

## 3. Experimental Section

### 3.1. Materials

Indium chloride tetrahydrate (InCl_3_·4H_2_O, AR) and copper chloride dihydrate (CuCl_2_·2H_2_O, AR) were purchased from Shanghai Aladdin Biochemical Technology Co., Ltd., Shanghai, China. Thioacetamide (TAA, AR) was provided by Sinopharm Chemical Reagent Co., Ltd., Shanghai, China. All reagents used during the experiment were as provided without further purification.

### 3.2. Synthesis of In_2_S_3_ and Vs-In_2_S_3_/CuInS_2_

The In_2_S_3_ crystals were prepared using a facile one-pot solvothermal method. Briefly, 37 mg of InCl_3_⋅4H_2_O and 15 mg of TAA were dissolved in 40 mL of ethanediol under ultrasonication. The mixture was then transferred into a hydrothermal autoclave (50 mL in capacity) and maintained at 150 °C for 90 min. The In_2_S_3_ microspheres were obtained by multiple centrifugations and thoroughly washed with water and ethanol, using a centrifugal speed of 4000 r/min and maintaining time of 1 min. Vs-In_2_S_3_/CuInS_2_ flower-like microspheres were subsequently synthesized using an In_2_S_3_ template-assisted and cation-exchange strategy. Typically, an appropriate amount of In_2_S_3_ was added to a 40 mL ethanediol solution containing CuCl_2_·2H_2_O and TAA under continuous stirring. The solution was then transferred to a sealed Teflon-lined autoclave and heated to 150 °C for 90 min. After the reaction was completed, the products were collected by centrifugation, washed several times with deionized water, and dried at 60 °C in a vacuum. The mass of CuCl_2_·2H_2_O was changed to 0.25, 0.5, 0.75, and 1.0 g, and the same procedures were performed to obtain Vs-In_2_S_3_/CuInS_2_ with different Cu masses; these products were denoted as 0.25-Vs-In_2_S_3_/CuInS_2_, 0.50-Vs-In_2_S_3_/CuInS_2_, 0.75-Vs-In_2_S_3_/CuInS_2_, and 0.10-Vs-In_2_S_3_/CuInS_2_, respectively.

### 3.3. Characterizations

X-ray diffraction (XRD, Rigaku D/MAX-Ultima III, Tokyo, Japan) was used to investigate the purity information and crystallographic phase of the as-prepared powder samples. The XRD pattern was recorded by using Cu-ka radiation (λ = 0.154178 nm) at 40 kV and 40 mA with a scan rate of 10° min^−1^. The morphology of nanomaterials was observed by field-emission scanning electron microscopy (FESEM, FEI NOVA NANOSEM 230). Transmission electron microscopy (TEM) and high-resolution TEM (HRTEM) images were captured using JEM 200CX TEM apparatus. Chemical states were investigated by X-ray photoelectron spectroscopy (XPS; K-Alpha, Thermo Fisher Scientific, Waltham, MA, USA); here, the spectra obtained were standardized according to the binding energy of the adventitious C 1s peak at 284.8 eV. A UV-vis spectrophotometer (UV-3600iPlus, Shimadzu, San Jose, CA, USA) was used to record the UV-visible diffuse reflectance of the samples. The sulfur vacancy signals were examined using spin-trapping electron paramagnetic resonance (EPR, EXM-10/12, Bruker, Bremen, Germany) measurements. The photoluminescence (PL) decay profiles of the samples were analyzed using single-particle confocal fluorescence spectroscopy measurements (PicoHarp300, Berlin, Germany).

### 3.4. Photoelectrochemical Measurements

Photoelectrochemical measurements were performed using electrochemical analysis (CHI-630D, Shanghai Chenhua, Shanghai, China) in a standard three-electrode system. The electrolyte was 0.5 M NaSO_4_ aqueous solution. The as-prepared In_2_S_3_ or Vs-In_2_S_3_/CuInS_2_ sample was used as the working electrode. A saturated Ag/AgCl electrode and a Pt foil were used as the reference and counter electrodes, respectively. The preparation process of In_2_S_3_ and Vs-In_2_S_3_/CuInS_2_ bulk samples was as follows. In_2_S_3_ or Vs-In_2_S_3_/CuInS_2_ catalyst ink was first prepared by dispersing 10 mg of the as-prepared catalyst in 1 mL of ethanol under sonication. Then, 50 μL of the ink was evenly spread onto a piece of pretreated fluorine-doped tin oxide (FTO) glass within a 1 cm^2^ area and dried at room temperature (25 °C). Thus, the catalysts were attached to the FTO surface. The transient photocurrent test curves exhibited ohmic characteristics, confirming the formation of an ohmic back contact between the sample and FTO. The working area of the electrode was 1 × 1 cm^2^, and the scan rate was 5 mV s^−1^. Electrochemical impedance spectroscopy (EIS) was performed using a PAR2273 workstation (Princeton Applied Research, USA) under AM 1.5G illumination. The Mott–Schottky curves of the samples were measured in 0.5 M Na_2_SO_4_ aqueous solution using an electrochemical workstation (CHI-630D, Shanghai Chenhua, Shanghai, China).

### 3.5. Photocatalytic Activity Measurements

Photocatalytic reduction of CO_2_ was performed on a photoreaction system. A 4–5 mg powder catalyst was dissolved under ultrasound for 10 min in the prepared solution, including 2 mL of deionized H_2_O and 1 mL of NaSO_3_ in a 460 mL reactor with a quartz glass cover. After the air in the reaction system was cleared away, high-purity CO_2_ was introduced into the system until the pressure reached 70 kPa and circulated for 60 min to achieve uniform distribution of CO_2_ gas. The temperature of photocatalytic CO_2_ reduction was set at 10 °C by cooling the water circulation system, which can promote the adsorption of CO_2_. A 300 W xenon arc lamp was used as the light source for the photocatalytic reaction. During irradiation, approximately 1 mL of gas was collected from the reaction cell at specific time intervals for CO and CH_4_ concentration analysis using a gas chromatograph (GC-2014C, Shimadzu Corp., Kyoto, Japan).

### 3.6. Density Functional Theory Calculation Details

The calculations are performed within the framework of density functional theory (DFT) using a basis set consisting of plane waves. The electron–ion interactions are described by ultrasoft pseudopotentials and electron exchange, and correlation energies are calculated with the Perdew–Burke–Ernzerhof (PBE) formulation of the generalized gradient approximation (GGA). The geometric structure is optimized with the Broyden–Fletcher–Goldfarb–Shanno (BFGS) method, the forces on each ion converge to less than 0.01 eV/Å, and the stress is less than 0.02 GPa. All the atoms of the structures are fully relaxed to their equilibrium positions with an energy convergence of 5 × 10^−6^ eV, the atomic displacement is less than 5 × 10^−4^ Å, and the self-consistent field (SCF) tolerance is 5 × 10^−7^ eV. The kinetic energy cutoff (In_2_S_3_: 350 eV; CuInS_2_: 440 eV) of the plane wave basis is used throughout, and the Brillouin zone is sampled with special k-points of a 3 × 3 × 1 grid for In_2_S_3_ and a 5 × 5 × 2 grid for CuInS_2_ based on Monkhorst–Pack.

## 4. Conclusions

In this study, Vs-In_2_S_3_/CuInS_2_ microflower heterojunctions were formed in situ using an In_2_S_3_ microsphere template-directed synthesis and metal ion exchange-mediated growth strategy. Hybrid photocatalysts with a flower-like microsphere structure were obtained using hydrothermally synthesized In_2_S_3_ microspheres as a template, followed by Ostwald ripening growth during the metal cation exchange of Cu^+^ and In^3+^. The as-fabricated Vs-In_2_S_3_/CuInS_2_ heterojunctions exhibited significantly improved photocatalytic activity for CO and CH_4_ evolution compared with pure In_2_S_3_ owing to the synergistic effects of enhancements in charge-carrier separation and visible-light absorption capacity. We believe that the findings could serve as guidance for future researchers aiming to engineer other metal sulfide-based photocatalysts with enhanced photocatalytic performance via a simple and efficient strategy.

## Data Availability

Data are contained within the article.

## Supplementary Materials

The following supporting information can be downloaded at: https://www.mdpi.com/article/10.3390/molecules29143334/s1, Figure S1: XPS survey spectrum of the prepared Vs-In_2_S_3_/CuInS_2_ composite film.
