# Supplementary figures and images for: Synthesis of Sulfur Vacancy-Bearing In2S3/CuInS2 Microflower Heterojunctions via a Template-Assisted Strategy and Cation-Exchange Reaction for Photocatalytic CO2 Reduction
Source: Molecules. 2024 Jul 16;29(14):3334. doi: 10.3390/molecules29143334 (PMC11279527; doi:10.3390/molecules29143334)

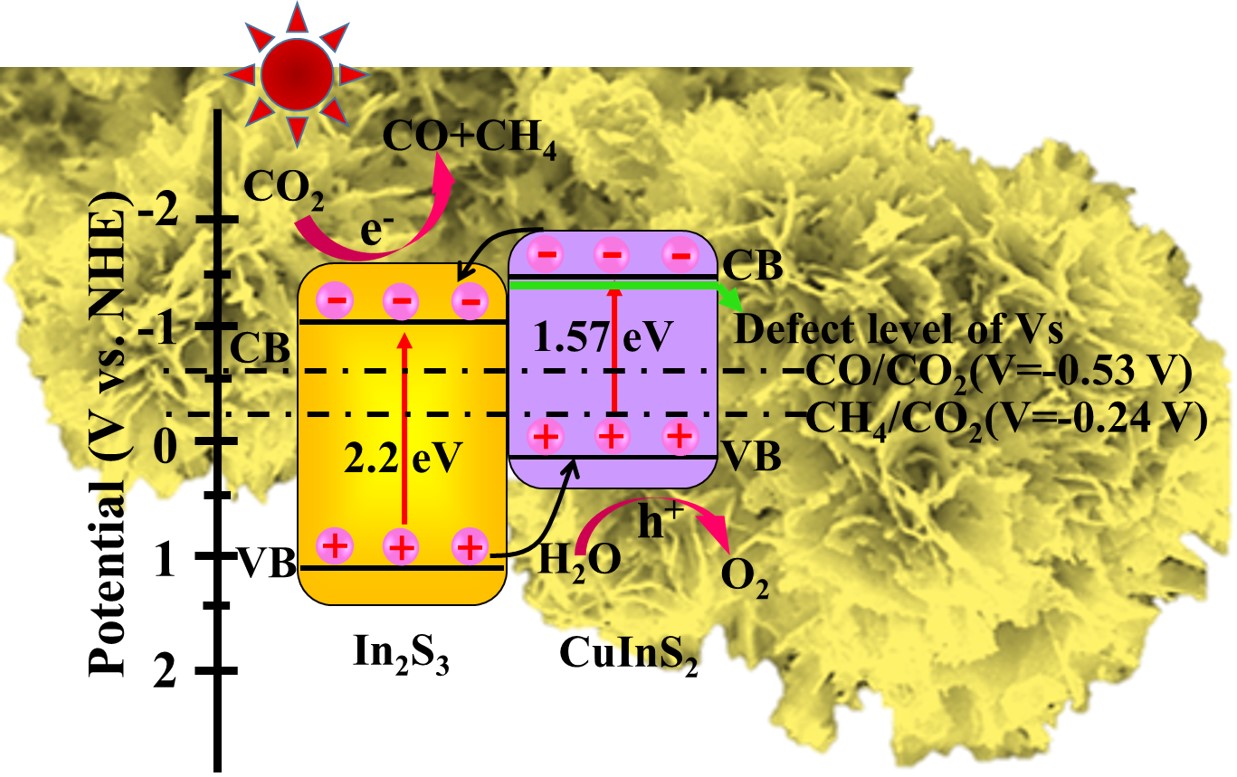

Supplement: Supplementary file 1 [file molecules-29-03334-s001.zip › molecules-3070467-supplementary.jpg]
